# Associations of the Philadelphia sweetened beverage tax with changes in adult body weight: an interrupted time series analysis

**DOI:** 10.1016/j.lana.2024.100906

**Published:** 2024-10-12

**Authors:** Joshua Petimar, Christina A. Roberto, Jason P. Block, Nandita Mitra, Emily F. Gregory, Emma K. Edmondson, Gary Hettinger, Laura A. Gibson

**Affiliations:** a Department of Population Medicine, Harvard Medical School & Harvard Pilgrim Health Care Institute, Boston, MA, USA; b Department of Epidemiology, Harvard T.H. Chan School of Public Health, Boston, MA, USA; c Department of Medical Ethics and Health Policy, Perelman School of Medicine, University of Pennsylvania, Philadelphia, PA, USA; d Department of Biostatistics, Epidemiology, & Informatics, Perelman School of Medicine, University of Pennsylvania, Philadelphia, PA, USA; e Children's Hospital of Philadelphia, Philadelphia, PA, USA

**Keywords:** Beverage tax, Body mass index, Obesity, Sugar-sweetened beverages, Nutrition policy, Food policy

## Abstract

**Background:**

Sweetened beverage taxes are associated with large decreases in sugar-sweetened beverage sales, but their effects on weight outcomes are unclear. We examined associations of the 2017 Philadelphia beverage tax with changes in adult weight outcomes.

**Methods:**

We obtained electronic health record data on adults 18–65 years old in Philadelphia (intervention) and other areas of Pennsylvania and New Jersey (control) from 2014 to 2019. Controlled interrupted time series models compared post-tax changes in trends of body mass index (BMI, primary outcome) and obesity prevalence (secondary outcome). A panel sample comprised 175,675 adults with at least one BMI measure in both the pre-tax (2014–2016) and post-tax (2017–2019) periods. A cross-sectional sample comprised 587,121 adults with at least one BMI measure from 2014 to 2019.

**Findings:**

Before tax implementation, Philadelphia panel patients had a mean BMI of 30.4 kg/m^2^ and an obesity prevalence of 44.5%. After implementation, in the panel sample, there was a −0.03 kg/m^2^ (95% CI: −0.07, 0.02) per quarter decrease in BMI vs. control, implying a −0.32 kg/m^2^ (−0.85, 0.20) change at the end of the 3-year study period. In the cross-sectional sample, there was a −0.05 kg/m^2^ (95% CI: −0.09, −0.01) per quarter decrease in BMI vs. control, implying a −0.60 kg/m^2^ (−1.04, −0.16) change at the end of the study period. Results for obesity prevalence were consistent with the BMI results.

**Interpretation:**

There was some limited evidence of a decrease in BMI and obesity prevalence in Philadelphia 3 years after beverage tax implementation. Replication of these results is needed.

**Funding:**

National Institutes of Health.

**Research in context**

**Evidence before this study**

We searched the PubMed database for studies evaluating the effect of sweetened beverage excise taxes on weight-related outcomes through December 31, 2022 (terms (beverage tax BMI) OR (beverage tax weight) OR (beverage tax obesity) OR (beverage tax health)). These were supplemented with Google Scholar searches and the authors’ own knowledge of beverage tax evaluation studies. Studies were restricted to those published in English. This strategy yielded 3 studies, all of which were conducted outside the U.S. (Mauritius, Mexico, South Africa). Two studies were conducted only among children, and another was conducted among all age groups. One study used a pre-post design without a control group, while the others used quasi-experimental designs. We repeated this search strategy during the revision stage (for papers published from January 1, 2023 through August 15, 2024) and identified 3 additional studies, all of which were conducted in the U.S. Two of those studies were conducted in children or adolescents and the other was conducted among pregnant individuals. To date, there is weak evidence for the effect of sweetened beverage taxes on weight-related outcomes, particularly in U.S. adult populations.

**Added value of this study**

The findings of this large, quasi-experimental study add to the growing literature on the impact of sweetened beverage excise taxes and health outcomes. Overall, this study found some limited evidence of decreases in BMI and obesity prevalence in adults living in Philadelphia after the beverage tax was implemented (compared to those living in control areas), with stronger and more precisely estimated results in a cross-sectional sample of patients than a longitudinal panel sample. This study therefore finds that sweetened beverage taxes may have weight-related health benefits for non-pregnant adults, which is a new contribution to the existing literature.

**Implications of all the available evidence**

The available evidence, including the present study, suggests that sweetened beverage excise taxes may yield improvements in weight-related outcomes, though the findings across studies are not consistent. The evidence base consists of an overall small number of studies conducted across different countries, age ranges, and specific health outcomes, which may explain the inconsistent findings. Additional studies are needed to confirm the effects of sweetened beverage taxes on weight-related outcomes, especially in adults.

## Introduction

Sugar-sweetened beverage (SSB) consumption is associated with adverse metabolic health outcomes, including obesity, type 2 diabetes, and heart disease.^1^ SSBs are the largest source of added sugar in the U.S. diet, comprising ∼25% of U.S. added sugar intake.^2^ Interventions that aim to reduce SSB consumption may therefore improve public health. One such intervention is sweetened beverage taxation, which has been implemented in >100 countries and several U.S. jurisdictions.^3^ In the U.S., these excise taxes, which range from 1 to 2 cents/oz, are associated with large reductions in SSB purchases^4^ and generate revenue that cities have invested in education, economic development, and community health initiatives.^5^

Tax-related declines in SSB purchases have the potential to improve health, and simulation studies have projected appreciable declines in body mass index (BMI) and prevalence of metabolic disease following beverage tax implementation.^6^^,^^7^ However, very few studies have evaluated the effect of beverage taxes on weight-related outcomes in adults. Two studies in children observed some small improvements in body weight in response to sweetened beverage taxes in Mexico^8^ and Seattle,^9^ though a separate study of Mauritius's beverage tax found no changes in youth BMI.^10^ A study using birth certificate data across large U.S. cities found small post-tax reductions in weight-for-gestational-age and risk of gestational diabetes and small-for-gestational-age births.^11^ To date, no studies have examined associations of U.S. beverage taxes on weight outcomes in non-pregnant adults.

This study examines the Philadelphia beverage tax, a 1.5-cent per ounce tax placed on both SSBs and artificially sweetened beverages that was implemented on January 1, 2017. Large quasi-experimental studies of the Philadelphia beverage tax have observed that this tax was associated with a mean 29–34% increase in beverage price and 20–40% declines in SSB purchases following implementation.^12^, ^13^, ^14^ These large reductions in sales suggest that the tax may further yield health benefits, particularly reductions in weight-related outcomes. We therefore examined associations of the Philadelphia beverage tax with changes in adult BMI and obesity prevalence in Philadelphia compared to control areas using electronic health record data and a controlled interrupted time series approach.

## Methods

### Study sample

We obtained electronic health record data from Penn Medicine, the University of Pennsylvania Health System, for residents of Pennsylvania and New Jersey from 2014 to 2019. Penn Medicine includes 6 hospitals, as well as outpatient practices in 27 counties across Pennsylvania and New Jersey, with approximately 7 million outpatient visits annually.^15^ Within Philadelphia, Penn Medicine serves as one of several safety-net health systems, particularly in West Philadelphia, which is a large, low-income area in the city. The data we received from Penn Medicine included information on patients' age, gender, race, ethnicity, insurance type, height, weight, hemoglobin A1c (HbA1c) values, and International Classification of Diseases (ICD-10) codes for type 2 diabetes. Data on residential zip codes identified patients living in Philadelphia (intervention) or in other areas of Pennsylvania and New Jersey (control). We also obtained data on patients’ residential census tract.

The electronic health record data included information from 857,823 adults aged 18 years or older who had a weight or height measure from January 1, 2014 through December 31, 2019 (n = 5,408,028 visits). We did not include visits in 2020 or later due to the COVID-19 pandemic. The dataset was limited to individuals who either lived only in Philadelphia or only in control areas over the study period to reduce treatment contamination (i.e., we excluded people who moved between areas). We excluded visits from individuals ≥65 years old (n = 1,713,225) because BMI is less predictive of metabolic health in older adults due to age-related declines in lean body mass. We excluded visits where weight or height data were missing (n = 162,461) or were flagged as erroneous by *growthcleanr*,^16^ an R package for cleaning height and weight data in electronic health records (n = 983,328). We also excluded visits missing data on residential zip code (n = 17) because we did not know whether these individuals lived in Philadelphia or control areas, as well as those missing data on ≥1 covariate (n = 269,790).

After applying all exclusions, the analytic dataset consisted of a cross-sectional sample of 587,121 patients (n = 2,062,301 visits). We further identified a longitudinal sample of 175,675 patients (n = 1,207,816 visits) who had height and weight data in both the pre-tax (2014–2016) and post-tax period (2017–2019). This panel sample included individuals who were 18–59 years old on January 1, 2014 (i.e., all patients were <65 years over the entire study period). Although the panel sample was less vulnerable to changes in population composition, we also analyzed the cross-sectional sample because it was broader and included patients who might have had more limited access to healthcare (and therefore might have benefitted more from the tax).

### Measures

The primary outcome was BMI, calculated as weight/height^2^ (kg/m^2^). We used *growthcleanr* to clean the height and weight data (see Supplementary Methods).^16^ To further reduce random measurement error in BMI after implementing *growthcleanr*, we used each individual's modal height for all BMI calculations (i.e., we assumed no substantial changes in height given that all individuals were 18–65 years over the study period). We aggregated BMI at the patient-quarter level; if patients had >1 BMI measure in a quarter, we calculated their average BMI. For each quarter, we classified patients' overweight/obesity status (healthy weight: BMI <25; overweight: 25 ≤BMI <30; obesity: BMI ≥30). Obesity prevalence (i.e., probability of obesity) was the secondary outcome. HbA1c concentration was an exploratory outcome and was examined only among those without evidence of diabetes pre-tax (see Supplementary Methods).

We calculated the Yost index score of each patients’ residential census tract as a measure of neighborhood-level socioeconomic status^17^ (see Supplementary Methods), and their Medicaid status as a measure of individual-level socioeconomic status (Medicaid is available to adults in Pennsylvania and New Jersey with income <138% of the federal poverty level). For patients in the panel only, we calculated the total number of visits from 2014 to 2016 as a measure of pre-tax healthcare utilization.

### Statistical analysis

We used controlled interrupted time series models to estimate changes in BMI trends in Philadelphia versus control patients. This approach assumed that, had the beverage tax not been implemented, the pre-tax BMI trend in Philadelphia would have continued in the post-tax period, less any pre-post changes in BMI trends that were also observed in the control group (to account for potential time-varying confounders common to both groups). Any difference between this estimated counterfactual BMI trend and the actual trend in BMI in the post-tax period was assumed to be attributable to the tax.^18^

We used directed acyclic graphs to identify covariates that could have influenced outcome trends and therefore could have led to confounding if their distributions differed between groups (Supplementary Figure S1). These included age, gender, race, Hispanic ethnicity, Medicaid status, Yost score, and, for panel analyses, number of pre-tax healthcare visits. We adjusted for these using inverse probability of treatment weights (IPTW). We weighted the control patients in each time interval such that their covariate distribution resembled that of Philadelphia in that time interval, thus estimating the average treatment effect among the treated^19^ (see Supplementary Methods for details of IPTW construction).

We estimated post-tax changes in BMI trends using IPTW-weighted generalized estimating equations with BMI as the response variable, an identity link, and assuming a normal distribution. We used cluster-robust standard errors to account for repeated outcome measures at the patient-level and assumed an independence correlation structure, which fit the data best. The model included terms for group (Philadelphia vs. control), time (continuous quarter), a group-by-time interaction (which allowed different pre-tax trends between groups), weeks-after-tax (i.e., the change in BMI trend for the control group following the tax), and an interaction term between group and weeks-after-tax, which was the coefficient of interest and estimated the post-tax change in BMI trend in Philadelphia less the trend change in the control group (“differenced trend change”). The model also adjusted for season. We used similar methods to estimate percentage point changes in obesity prevalence trends. We conducted analyses separately for the panel and cross-sectional samples.

We conducted stratified analyses by gender (men vs. women), hypothesizing greater tax-related decreases in obesity prevalence among women, given observed reductions in obesity prevalence among girls only in Mexico.^8^ We stratified by race (White vs. Black) given some evidence that SSBs comprise a higher proportion of added sugar intake among Black vs. White populations^20^; we therefore predicted stronger associations among Black vs. White patients. We did not examine associations by Hispanic ethnicity owing to small sample sizes. In the panel only, we stratified by weight status (healthy weight vs. overweight vs. obesity), hypothesizing larger reductions in weight among those with baseline obesity because they tend to consume more SSBs than those without obesity.^21^ Lastly, in cross-sectional analyses, we stratified by Medicaid status because lower-income patients might be more sensitive to tax-related price increases and reduce SSB consumption more than higher-income patients.

We conducted several sensitivity analyses. First, we aggregated data at the patient-month level to determine robustness to estimating changes in monthly vs. quarterly trends. Second, we excluded the first 6 months of the post-tax period. Our previous study found a strong and immediate change in beverage sales following tax implementation,^12^ suggesting that changes in weight could have begun soon after as well, but it is also possible that the effect of the tax on body weight could have been delayed. Third, we removed BMI values that were outliers even after running *growthcleanr* (>3 standard deviations above the mean, affecting <1% of patients). Lastly, for panel analyses only, we restricted to individuals with BMI measures every year of the study to further reduce possible bias due to changing population composition.

We examined changes in HbA1c concentration among all patients without diabetes pre-tax overall and separately among those who had pre-diabetes (5.7% ≤ HbA1c ≤6.4%). Because we did not have information on diabetes medications or laboratory measures besides HbA1c, diabetes status could have been misclassified.^22^ These analyses were therefore considered exploratory.

We conducted analyses with SAS version 9.4 (Cary, NC) and calculated 2-sided 95% confidence intervals (CI). This study was approved by the University of Pennsylvania Institutional Review Board. We did not obtain consent from participants prior to study commencement. Ethical approval was not required because the Institutional Review Board deemed that there were adequate provisions to protect the privacy of subjects and to maintain the confidentiality of the data.

## Results

At baseline, the median (interquartile range) age of Philadelphia patients in the panel sample was 42.0 years old (30.8–51.0), and the sample was 41% male, 59% female, 41% White, 50% Black, 4% Asian American or Pacific Islander, 5% other or multiple races, and 4% Hispanic ethnicity (Table 1). Most Philadelphia panel patients lived in neighborhoods with lower socioeconomic status (65% had a Yost index score of 1 or 2) and 20% of their visits were paid with Medicaid benefits. Compared to Philadelphia, control patients were more likely to be White (83%) and less likely to be Black (9%), pay with Medicaid benefits (3%), or live in low-socioeconomic status neighborhoods (6% had a Yost score of 1 or 2). After applying IPTW, covariate balance improved greatly, and there were few differences between groups. This was also true of the cross-sectional sample overall (Table 1) and across intervention periods (Supplementary Table S1). The mean (standard deviation) of the IPTWs was 0.62 (1.33) for the panel sample and 0.61 (1.25) for the cross-sectional sample.

**Table 1:** Characteristics of the panel and cross-sectional datasets before and after inverse probability of treatment weighting.

Characteristic^a^	Unweighted		Weighted^b^	
	Philadelphia	Control	Philadelphia	Control
**Panel sample**				
Age at baseline (y)	42.0 (30.8, 51.0)	45.8 (35.6, 52.0)	42.0 (30.8, 51.0)	40.9 (29.8, 50.0)
No. pre-tax visits	4.0 (2.0, 8.0)	4.0 (2.0, 7.0)	4.0 (2.0, 8.0)	4.0 (2.0, 8.0)
Gender				
Male	22,400 (41%)	49,636 (41%)	22,400 (41%)	22,826 (42%)
Female	32,553 (59%)	71,086 (59%)	32,553 (59%)	31,819 (58%)
Race				
White	22,484 (41%)	99,713 (83%)	22,484 (41%)	23,044 (42%)
Black	27,267 (50%)	10,785 (9%)	27,267 (50%)	25,974 (48%)
Asian-American/Pacific Islander	2376 (4%)	4720 (4%)	2376 (4%)	2630 (5%)
Other/multiple	2826 (5%)	5504 (5%)	2826 (5%)	2997 (5%)
Hispanic ethnicity	2173 (4%)	3793 (3%)	2173 (4%)	23,320 (4%)
Medicaid participation	10,755 (20%)	4066 (3%)	10,755 (20%)	10,120 (19%)
Yost Quintile				
1	24,224 (44%)	2857 (2%)	24,224 (44%)	23,879 (44%)
2	11,305 (21%)	4398 (4%)	11,305 (21%)	11,234 (21%)
3	7438 (14%)	14,209 (12%)	7438 (14%)	7498 (14%)
4	6996 (13%)	28,849 (24%)	6996 (13%)	7026 (13%)
5	4990 (9%)	70,409 (58%)	4990 (9%)	5008 (9%)
**Cross-sectional sample**				
Age at BMI measurement (y)	46.7 (33.6, 56.5)	49.9 (37.8, 57.8)	46.7 (33.6, 56.5)	45.3 (32.5, 55.2)
Gender				
Male	256,339 (41%)	594,373 (42%)	256,339 (41%)	257,297 (41%)
Female	374,480 (59%)	837,109 (58%)	374,480 (59%)	374,480 (59%)
Race				
White	246,614 (39%)	1,168,361 (82%)	246,614 (39%)	253,736 (41%)
Black	323,456 (51%)	133,861 (9%)	323,456 (51%)	303,368 (49%)
Asian-American/Pacific Islander	29,221 (5%)	60,322 (4%)	29,221 (5%)	32,301 (5%)
Other/multiple	31,528 (5%)	68,938 (5%)	31,528 (5%)	32,738 (5%)
Hispanic ethnicity	26,614 (4%)	49,158 (3%)	26,614 (4%)	28,609 (5%)
Medicaid	144,210 (23%)	58,529 (4%)	144,210 (23%)	137,120 (22%)
Yost Quintile				
1	291,781 (46%)	39,229 (3%)	291,781 (46%)	284,172 (46%)
2	128,385 (20%)	57,787 (4%)	128,385 (20%)	126,013 (20%)
3	82,888 (13%)	176,684 (12%)	82,888 (13%)	83,299 (13%)
4	74,511 (12%)	351,780 (25%)	74,511 (12%)	75,083 (12%)
5	53,254 (8%)	806,002 (56%)	53,254 (8%)	53,475 (9%)

a Median (interquartile range) or N (%).

b The sample size for the control group in the weighted analysis was lower than in the unweighted analysis because the the mean IPTW was <1 for the control group.

**Fig. 1:**
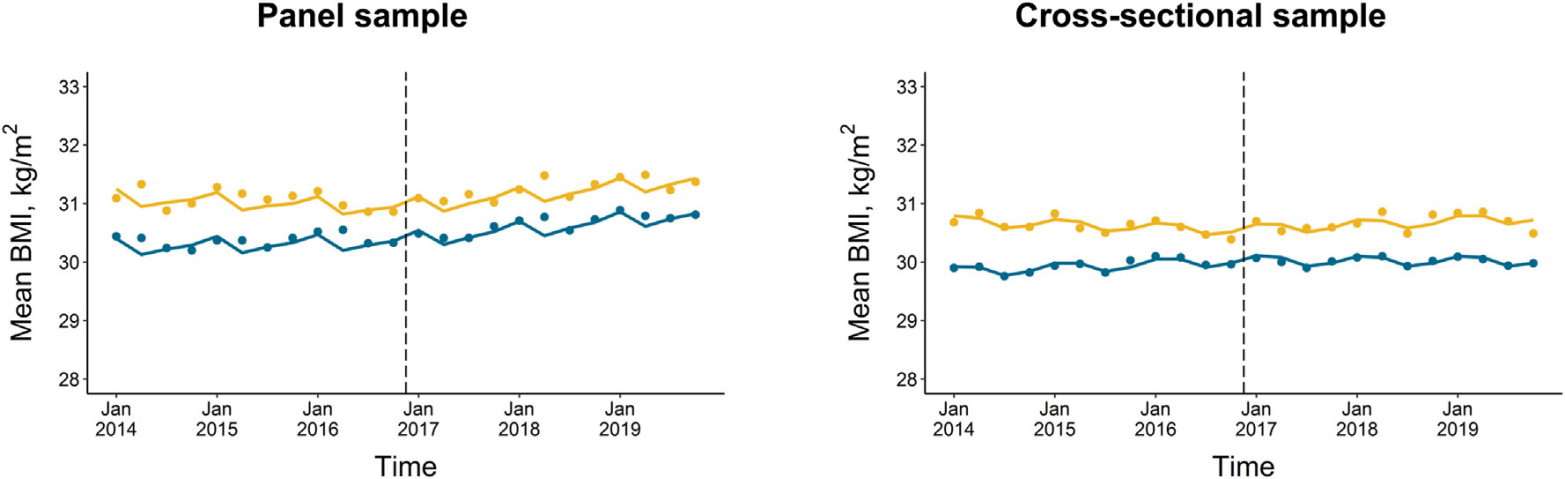
**Changes in BMI trends in Philadelphia and control patients after Philadelphia sweetened beverage tax implementation.** The figure shows the mean BMI in each quarter after inverse probability of treatment weighting (dots) and the predicted BMI from weighted CITS models (solid lines) for Philadelphia patients (blue) and control patients (gold). The dashed vertical line in January 2017 represents the date of tax implementation.

In the panel sample, the weighted models estimated that in the pre-tax period, the mean BMI was 30.4 kg/m^2^ (95% CI: 30.3, 30.5) in Philadelphia patients and 31.3 kg/m^2^ (30.9, 31.6) in control patients. Pre-tax BMI trends appeared flat in both Philadelphia (trend = 0.01 kg/m^2^ [−0.01, 0.02]) and control (trend = −0.02 kg/m^2^ [−0.04, 0.01]). After the tax went into effect, the BMI trend increased in Philadelphia by 0.03 kg/m^2^ per quarter (0.02, 0.05) and in the control group by 0.06 kg/m^2^ per quarter (0.02, 0.10), yielding a differenced trend change of −0.03 kgm^2^ per quarter (−0.07, 0.02) (Table 2, Fig. 1). This differenced trend change implies an estimated change in BMI of −0.32 kg/m^2^ (−0.85, 0.20) at the end of the 3-year study period. The panel BMI results were generally similar across population subgroups, though associations were stronger in Black patients (differenced trend change = −0.07 kgm^2^ [−0.15, 0.00]) than White patients (differenced trend change = 0.03 kgm^2^ [−0.01, 0.06]). The main panel BMI results were similar in most sensitivity analyses but were attenuated when restricting to those with BMI measures in every year of the study period (differenced trend change = 0.01 kgm^2^ [−0.06, 0.08]) (Supplementary Table S2). Supplementary Figure S2 displays the BMI distribution for Philadelphia and control patients in the pre- and post-tax periods, weighted by IPTWs.

**Table 2:** Changes in BMI trends^a^ after Philadelphia beverage tax implementation.

Sample	Control			Philadelphia			Differenced trend change in BMI^c^	P-value^d^
	Baseline BMI^b^	Pre-tax BMI trend	Post-tax BMI trend change	Baseline BMI^b^	Pre-tax BMI trend	Post-tax BMI trend change		
**Panel sample**^e^								
Overall	31.3 (30.9, 31.6)	−0.02 (−0.04, 0.01)	0.06 (0.02, 0.10)	30.4 (30.3, 30.5)	0.01 (−0.01, 0.02)	0.03 (0.02, 0.05)	−0.03 (−0.07, 0.02)	0.23
Men	30.9 (30.4, 31.3)	−0.02 (−0.06, 0.02)	0.06 (0.00, 0.12)	29.6 (29.4, 29.7)	0.01 (0.00, 0.03)	0.01 (−0.01, 0.04)	−0.05 (−0.11, 0.01)	0.12
Women	31.6 (31.1, 32.1)	−0.01 (−0.05, 0.03)	0.05 (0.00, 0.11)	30.9 (30.7, 31.0)	0.01 (0.00, 0.02)	0.04 (0.02, 0.06)	−0.02 (−0.08, 0.04)	0.57
White race	29.3 (29.0, 29.5)	0.04 (0.02, 0.06)	−0.02 (−0.05, 0.01)	27.5 (27.3, 27.6)	0.03 (0.02, 0.04)	0.00 (−0.02, 0.03)	0.03 (−0.01, 0.06)	0.15
Black race	33.5 (32.9, 34.2)	−0.04 (−0.09, 0.00)	0.09 (0.01, 0.17)	32.6 (32.5, 32.8)	0.02 (0.01, 0.03)	0.02 (0.00, 0.04)	−0.07 (−0.15, 0.00)	0.063
Baseline healthy weight	22.2 (22.0, 22.3)	0.03 (0.01, 0.04)	0.05 (0.02, 0.07)	22.2 (22.1, 22.2)	0.02 (0.02, 0.03)	0.04 (0.03, 0.05)	−0.01 (−0.03, 0.02)	0.54
Baseline overweight	27.3 (27.2, 27.5)	0.04 (0.02, 0.05)	0.01 (−0.01, 0.03)	27.3 (27.3, 27.4)	0.03 (0.03, 0.04)	0.01, (0.00, 0.02)	0.00 (−0.03, 0.02)	0.81
Baseline obesity	37.6 (37.1, 38.1)	0.00 (−0.04, 0.04)	−0.01 (−0.07, 0.05)	37.2 (37.0, 37.3)	0.03 (0.02, 0.04)	−0.04 (−0.06, −0.02)	−0.03 (−0.09, 0.03)	0.35
**Cross-sectional sample**^f^								
Overall	30.7 (30.4, 30.9)	−0.01 (−0.04, 0.01)	0.03 (0.00, 0.07)	29.8 (29.7, 29.9)	0.02 (0.01, 0.03)	−0.02 (−0.03, −0.01)	−0.05 (−0.09, −0.01)	0.0073
Men	30.4 (30.0, 30.8)	−0.02 (−0.06, 0.01)	0.03 (−0.02, 0.08)	29.0 (28.9, 29.1)	0.02 (0.01, 0.03)	−0.02 (−0.04, 0.00)	−0.05 (−0.10, 0.01)	0.093
Women	30.9 (30.6, 31.2)	0.00 (−0.04, 0.03)	0.03 (−0.01, 0.08)	30.3 (30.1, 30.4)	0.02 (0.01, 0.03)	−0.02 (−0.03, 0.00)	−0.05 (−0.10, 0.00)	0.046
White race	29.2 (29.0, 29.3)	0.01 (0.00, 0.03)	0.00 (−0.02, 0.02)	27.4 (27.3, 27.5)	0.02 (0.01, 0.03)	−0.01 (−0.02, 0.01)	−0.01 (−0.04, 0.02)	0.61
Black race	32.6 (32.2, 33.1)	−0.03 (−0.07, 0.01)	0.06 (0.00, 0.13)	32.1 (31.9, 32.2)	0.02 (0.01, 0.03)	0.00 (−0.02, 0.02)	−0.07 (−0.13, 0.00)	0.047
Medicaid	32.4 (31.5, 33.3)	−0.09 (−0.17, −0.01)	0.10 (−0.02, 0.21)	31.6 (31.4, 31.9)	−0.04 (−0.06, −0.02)	0.04 (0.00, 0.07)	−0.06 (−0.18, 0.06)	0.30
Non-Medicaid	30.3 (30.1, 30.5)	0.00 (−0.02, 0.02)	0.02 (−0.01, 0.05)	29.3 (29.2, 29.4)	0.02 (0.01, 0.03)	−0.02 (−0.03, 0.00)	−0.04 (−0.07, −0.01)	0.014

a Models were fit with generalized estimating equations weighted by inverse probability of treatment weights with variables for group (Philadelphia = 1, control = 0), time (continuous quarter), trend change (i.e., continuous quarter since tax implementation), a group-by-time interaction, a group-by-trend change interaction, and indicator terms for season. We estimated the baseline BMI level and pre-tax trend in BMI in Philadelphia using the same model but reversing the coding of the Philadelphia and control group.

b Mean BMI in the first quarter of 2014.

c Post-tax change in BMI trend in Philadelphia, less that of the control group.

d P-value for the differenced trend change.

e The number of patients included in the panel analysis: overall: n = 175,675; men: n = 72,036; women: n = 103,639; White race: n = 122,197; Black race: n = 38,052; baseline healthy weight: n = 58,000; baseline overweight: n = 56,475; baseline obesity: n = 61,200.

f The number of patients included in the cross-sectional analysis: overall: n = 587,121; men: n = 254,859; women: n = 332,262; White race: n = 409,726; Black race: n = 113,211; Medicaid: n = 65,907; non-Medicaid: n = 536,750.

We observed slightly stronger associations for BMI in the cross-sectional sample (Table 2, Fig. 1). Among all cross-sectional patients, there was a differenced trend change of −0.05 kg/m^2^ per quarter (−0.09, −0.01), implying a −0.60 kg/m^2^ (−1.04, −0.16) decrease at the end of the study period. Associations were again slightly stronger in Black patients (differenced trend change = −0.07 kgm^2^ [−0.13, 0.00]) than White patients (differenced trend change = −0.01 kgm^2^ [−0.04, 0.02]). Results were generally similar in sensitivity analyses (Supplementary Table S2).

We observed a similar pattern of results for obesity prevalence (Table 3). Among all panel patients, there was a differenced trend change of −0.2 percentage points (−0.5, 0.0) per quarter, with slightly stronger associations among Black patients (differenced trend change = −0.4 percentage points [−0.9, 0.0]) than White patients (differenced trend change = 0.0 percentage points [−0.2, 0.2]). In the cross-sectional sample, we observed a post-tax decline in obesity prevalence of −0.3 percentage points (−0.5, 0.0) per quarter, with again slightly stronger associations in Black (differenced trend change = −0.4 percentage points [−0.8, 0.0]) vs. White patients (differenced trend change = 0.0 percentage points [−0.2, 0.2]). Results for obesity prevalence were similar in sensitivity analyses (Supplementary Table S3).

**Table 3:** Changes in obesity prevalence trends^a^ after Philadelphia beverage tax implementation.

Sample	Control			Philadelphia			Differenced trend change in obesity prevalence^c^	P-value^d^
	Baseline obesity prevalence^b^	Pre-tax trend in obesity prevalence	Post-tax trend change in obesity prevalence	Baseline obesity prevalence^b^	Pre-tax trend in obesity prevalence	Post-tax trend change in obesity prevalence		
**Panel sample**^e^								
Overall	49.8% (47.9, 51.7)	−0.1pp (−0.3, 0.0)	0.4pp (0.2, 0.7)	44.5% (43.8, 45.3)	0.0pp (0.0, 0.1)	0.2pp (0.1, 0.3)	−0.2pp (−0.5, 0.0)	0.089
Men	48.1% (45.2, 51.0)	−0.2pp (−0.5, 0.1)	0.6pp (0.2, 1.0)	38.5% (37.3, 39.7)	0.1pp (0.0, 0.2)	0.0pp (−0.1, 0.2)	−0.5pp (−0.9, −0.1)	0.019
Women	51.3% (48.7, 53.8)	−0.1pp (−0.3, 0.1)	0.4pp (0.0, 0.7)	48.0% (47.0, 49.0)	0.0pp (−0.1, 0.1)	0.3pp (0.2, 0.4)	−0.1pp (−0.4, 0.3)	0.63
White race	39.0% (37.5, 40.4)	0.2pp (0.0, 0.3)	0.0pp (−0.2, 0.2)	26.9% (25.8, 27.9)	0.2pp (0.1, 0.3)	0.0pp (−0.1, 0.2)	0.0pp (−0.2, 0.2)	0.97
Black race	62.0% (58.6, 65.5)	−0.3pp (−0.6, 0.0)	0.6pp (0.1, 1.0)	58.2% (57.1, 59.2)	0.1pp (0.0, 0.2)	0.2pp (0.0, 0.3)	−0.4pp (−0.9, 0.0)	0.066
**Cross-sectional sample**^f^								
Overall	46.0% (44.7, 47.3)	−0.1pp (−0.2, 0.1)	0.2pp (0.0, 0.4)	40.9% (40.4, 41.5)	0.1pp (0.0, 0.1)	−0.1pp (−0.2, 0.0)	−0.3pp (−0.5, 0.0)	0.018
Men	43.7% (41.7, 45.8)	−0.1pp (−0.3, 0.1)	0.1pp (−0.2, 0.5)	35.2% (34.3, 36.1)	0.1pp (0.0, 0.2)	−0.1pp (−0.3, 0.0)	−0.3pp (−0.6, 0.1)	0.13
Women	47.4% (45.7, 49.1)	0.0pp (−0.2, 0.1)	0.2pp (−0.1, 0.4)	44.6% (43.9, 45.4)	0.1pp (0.0, 0.1)	−0.1pp (−0.2, 0.0)	−0.3pp (−0.5, 0.0)	0.082
White race	37.8% (36.7, 38.6)	0.1pp (0.0, 0.2)	−0.1pp (−0.2, 0.1)	26.8% (26.0, 27.6)	0.1pp (0.0, 0.2)	−0.1pp (−0.2, 0.1)	0.0pp (−0.2, 0.2)	0.97
Black race	57.7% (55.3, 60.1)	−0.2pp (−0.5, 0.00)	0.4pp (0.0, 0.8)	54.7% (53.8, 55.6)	0.1pp (0.0, 0.2)	0.0pp (−0.1, 0.1)	−0.4pp (−0.8, 0.0)	0.044
Medicaid	54.1% (49.5, 58.6)	−0.5pp (−0.9, 0.0)	0.5pp (−0.2, 1.1)	51.3% (49.8, 52.8)	−0.3pp (−0.4, −0.2)	0.3pp (0.1, 0.5)	−0.2pp (−0.8, 0.6)	0.68
Non-Medicaid	44.4% (43.2, 45.6)	0.0pp (−0.1, 0.1)	0.1pp (−0.1, 0.3)	38.6% (38.0, 39.2)	0.1pp (0.1, 0.2)	−0.1pp (−0.2, 0.0)	−0.3pp (−0.5, −0.1)	0.012

a Models were fit with generalized estimating equations weighted by inverse probability of treatment weights with variables for group (Philadelphia = 1, control = 0), time (continuous quarter), trend change (i.e., continuous quarter since tax implementation), a group-by-time interaction, a group-by-trend change interaction, and indicator terms for season. Baseline obesity prevalence is reported as a percent for each location. All trends and trend changes are reported as percentage points (pp). We estimated the baseline obesity prevalence level and pre-tax trend in obesity prevalence in Philadelphia using the same model but reversing the coding of the Philadelphia and control group.

b Obesity prevalence in the first quarter of 2014.

c Post-tax change in obesity prevalence trend in Philadelphia, less that of the control group.

d P-value for the differenced trend change.

e The number of patients included in the panel analysis: overall: n = 175,675; men: n = 72,036; women: n = 103,639; White race: n = 122,197; Black race: n = 38,052; baseline healthy weight: n = 58,000; baseline overweight: n = 56,475; baseline obesity: n = 61,200.

f The number of patients included in the cross-sectional analysis: overall: n = 587,121; men: n = 254,859; women: n = 332,262; White race: n = 409,726; Black race: n = 113,211; Medicaid: n = 65,907; non-Medicaid: n = 536,750.

In exploratory analyses in the panel sample, we did not observe associations of the tax with HbA1c concentrations overall (differenced trend change = −0.01% [−0.02, 0.00]) or when restricting to those with pre-diabetes (differenced trend change = 0.00% [−0.02, 0.01]) (Supplementary Table S4).

## Discussion

This study found some limited evidence of a reduction in body weight in adults living in Philadelphia after beverage tax implementation compared to adults living in control areas. After tax implementation, there was a statistically non-significant trend change in BMI of −0.03 kg/m^2^ per quarter in the panel sample, equivalent to −0.32 kg/m^2^ at the end of the 3-year study period, and a statistically significant trend change of −0.05 kg/m^2^ per quarter in the cross-sectional sample, equivalent to −0.60 kg/m^2^ at the end of the study period. The magnitude of these 3-year estimates implies that the tax has the potential to save tens of thousands of life-years and billions of dollars in healthcare costs if effects are sustained in the long-term.^6^ However, the generally wide 95% CIs, which crossed the null for the panel sample, undermine certainty in these estimates. Similarly, the panel sample suggested a statistically non-significant decline in obesity prevalence of −0.2 percentage points per quarter, while the cross-sectional sample yielded a statistically significant association of −0.3 percentage points per quarter. Despite the fact that many individual analyses were imprecisely estimated, when examined *overall*, the consistent results across study designs and outcomes point to a possible reduction in adult body weight in response to the Philadelphia beverage tax.

The Philadelphia beverage tax may have led to reduced body weight through its substantial effects on SSB purchases. Several independent studies have demonstrated 20–40% declines in taxed beverage purchases after tax implementation,^12^, ^13^, ^14^ even after accounting for increased shopping across the city border, which is larger than what has been observed for beverage taxes in other U.S. and global jurisdictions.^4^ One large study in adolescents found that the Philadelphia tax was associated with reduced SSB intake,^23^ though smaller consumption studies have found mixed results.^24^^,^^25^ We observed some slightly stronger associations between the tax and reduced BMI for Black compared to White patients, which we hypothesized given evidence that Black individuals may consume a greater proportion of total added sugars from SSBs than White individuals.^20^ A sweetened beverage tax would therefore be expected to be more effective among Black than White patients. We did not find differences in associations between those who did and did not pay for their visit with Medicaid benefits (a marker of socioeconomic status), contrary to a previous study that found stronger associations for dental outcomes among Medicaid participants.^26^ We also did not find differences in associations between men and women, despite some evidence that the tax has stronger effects in girls than boys.^8^ A study of Seattle's tax did not observe differences in associations between boys and girls.^9^

Although the panel and cross-sectional samples together point to tax-related reductions in weight-related outcomes, their individual results differed slightly, which may be explained by some differences in the composition of the two samples. The panel sample included patients with BMI measures in both the pre- and post-tax periods, which made it more robust to changes in population composition. The cross-sectional sample by contrast additionally included patients with measures in just one period, which could have made it more vulnerable to bias. However, we minimized this risk by creating IPTWs such that the distribution of covariates was similar between Philadelphia and control in every time interval, leading to no major differences between the two groups over time. The cross-sectional sample was also much larger and broader, which not only allowed for greater power to detect associations but also might have included a more diverse population. For example, patients who did not regularly seek healthcare (who may have had lower socioeconomic status) may not have had BMI measures in both periods and were therefore included only in the cross-sectional sample. The cross-sectional sample results therefore may be more generalizable than the panel. Despite these differences, results from both samples demonstrated small post-tax declines in BMI and obesity prevalence, though with different precision.

Although the overall evidence on the Philadelphia beverage tax suggests it improves diet and health outcomes, future taxes can be designed in ways that might further increase their effectiveness. For example, a tax can also be placed on liquid and powder beverage concentrates, which can be reconstituted with water to create SSBs. These products were not subject to the tax in Philadelphia and our analysis of 504 large retailers in Philadelphia and control areas demonstrated an increase in sales of these concentrates after the tax was implemented (though it is difficult to estimate the degree to which this substitution offset reductions in SSB purchases).^12^ Additionally, all U.S. beverage taxes are implemented at the city level, allowing residents to avoid the tax by purchasing SSBs in nearby neighboring jurisdictions. A sweetened beverage tax at the state level (similar to taxation of tobacco products) or federal level would reduce the amount of cross-border shopping and likely increase the impact of the tax.

This is the first study to evaluate a U.S. beverage tax with weight outcomes in non-pregnant adults. U.S. beverage taxes have been associated with lower weight gain-for-gestational-age z-score and reduced risk of gestational diabetes in pregnant adults.^11^ However, the tax's health effects may be more easily detected during pregnancy, when women may be motivated to make greater behavioral changes to support their health or the health of the fetus. A study of Seattle's sweetened beverage tax observed a 0.9-percentage point reduction in children's BMI (expressed as a percentage of the 95th percentile),^9^ and a study of Mexico's tax observed a 1.3-percentage point decline in obesity prevalence in girls (but not boys) per 10% increase in SSB prices.^8^ However, results from the Mexico study are difficult to compare with that of the present study because Mexico's 1 peso-per-liter tax was implemented alongside an 8% sales tax on nonessential foods high in fat, sodium, and added sugars,^27^ and the tax was nationwide, offering limited opportunity for tax avoidance. A separate study observed 22–24% reductions in tooth decay among older children and adults with lower income,^26^ highlighting the potential for the tax to improve oral health in addition to metabolic health.

This study has limitations. First, before applying IPTWs, there were large differences between Philadelphia and control patients on race, Medicaid status, and neighborhood socioeconomic status, which could lead to confounding. After applying IPTWs, the groups were balanced on all of these covariates. However, the large unadjusted differences between groups may have led to larger IPTWs (i.e., the weights had to be higher to achieve proper balance). This likely reduced statistical power and led to imprecise estimates, despite having a very large sample size. We might have had greater power to detect associations with a sample that was more balanced between Philadelphia and control (pre-weighting), or with an even larger sample size. It is also possible that Philadelphia and control patients differed on other factors that were not measured and therefore could not be adjusted for. Second, there may have been treatment contamination if patients living in one area commuted to the other area (e.g., for work, tax avoidance), which could have attenuated the results. Third, we did not have information on medications, which prevented us from identifying diabetes cases using standard electronic health record algorithms^22^ and fully accounting for bias in HbA1c analyses. Fourth, if there were other population health interventions (e.g., community obesity-prevention programs) during the study period that were implemented in only one of the groups, this could have affected outcome trends. We did not identify any major co-interventions that meet these criteria, but the presence and potential effects of such interventions cannot be completely ruled out. Lastly, because the electronic health records in the current study did not include information on SSB intake, our estimates were averaged over both SSB consumers and non-consumers. We might have found stronger evidence for these associations had we been able to estimate effects among only pre-tax SSB consumers, who likely experienced greater health benefits from the tax.

This study found some limited evidence of reduced BMI and obesity prevalence among adults living in Philadelphia compared to control areas following implementation of the Philadelphia beverage tax. If true, these findings imply further health benefits of the tax for reducing risk of nutrition-related chronic disease. However, confirmation of these findings is needed given that we observed slightly different results across different samples, leading to some uncertainty about the precise magnitude of the associations. Despite some uncertainty of the tax's effects on body weight, it has implied health benefits through large reductions in SSB purchases,^12^, ^13^, ^14^ which, if translated to reduced consumption, could lead to reduced chronic disease risk and mortality, even independent of body weight.^28^, ^29^, ^30^ The revenue the tax has raised for education, economic development, and community health initiatives will also likely improve long-term public health.^5^

## Contributors

CAR, EFG, EKE, and LAG were responsible for study conceptualization. JP and CAR were responsible for investigation. LAG was responsible for project administration. JP, CAR, JPB, NM, GH, and LAG were responsible for methodology. JP was responsible for data curation, formal analysis, visualization, and writing the original draft. CAR and LAG were responsible for supervision. CAR, JPB, NM, EFG, EKE, and LAG were responsible for funding acquisition. JP and LAG directly accessed and verified the underlying data. All authors reviewed edited, and approved the final version of the manuscript, and were responsible for the decision to submit the manuscript.

## Data sharing statement

No data or documents from this study will be shared. Electronic health record data from Penn Medicine are proprietary and the institution does not permit sharing of patient data with outside investigators.

## Declaration of interests

The authors declare no conflict of interest.

## Appendix A Supplementary data

Supplementary Methods, Tables, Figures and References
