# Supplementary material for: Associations of the Philadelphia sweetened beverage tax with changes in adult body weight: an interrupted time series analysis
Source: Lancet Reg Health Am. 2024 Oct 12;39:100906. doi: 10.1016/j.lana.2024.100906 (PMC11577562; doi:10.1016/j.lana.2024.100906)
Supplement: Supplementary Methods, Tables, Figures and References [file mmc1.pdf]

Supplementary Material

Associations of the Philadelphia sweetened beverage tax with changes in adult body weight: an interrupted time series analysis (Petimar et al.)

Joshua Petimar, Christina A. Roberto, Jason P. Block, Nandita Mitra, Emily F. Gregory, Emma K. Edmondson, Gary Hettinger, Laura A. Gibson

Supplementary Methods. Supplementary methodological details
Supplementary Table 1. Characteristics of the cross-sectional dataset by group and period before and after inverse probability of treatment weighting
Supplementary Table 2. Sensitivity analyses for changes in BMI trends after Philadelphia beverage tax implementation
Supplementary Table 3. Sensitivity analyses for changes in obesity prevalence trends after Philadelphia beverage tax implementation
Supplementary Table 4. Changes in HbA1c trends after Philadelphia beverage tax implementation
Supplementary Figure 1. Directed acyclic graph for the association between the Philadelphia beverage tax and change in adult BMI
Supplementary Figure 2. Differences in the distribution of BMI from pre- to post-tax for Philadelphia and control patients (weighted by IPTWs)

Supplementary Methods

1. *growthcleanr* for cleaning height and weight data.

growthcleanr uses an algorithm to remove biologically implausible values and values that are the result of common errors found in EHRs, including swapped height and weight measures, carry-forward values, same-day duplicates, and values recorded in the wrong unit. *growthcleanr* has demonstrated high validity for cleaning pediatric anthropometric data compared to physician judgment and other cleaning algorithms.[1,2] It is currently the only available algorithm for cleaning adult anthropometric data from EHRs, though it has not yet been validated in this population.

2. HbA1c measures and population.

In exploratory analyses of HbA1c concentration, we excluded HbA1c values that were <4.0%, >20.0%, or missing (0.3% of all HbA1c observations). If there was >1 HbA1c measure in a given quarter for a given patient, we took the average for that quarter. These analyses were conducted only among those without evidence of diabetes before tax implementation. Patients were classified as having evidence of diabetes if they had either an ICD-10 code for diabetes (ICD-10 codes 08, 09, 10, 11, or 13) or an HbA1c value >6.4%. Those without a diabetes-related ICD-10 code and who had an HbA1c value ≤6.4% were classified as not having evidence of diabetes.

3. Yost Index.

The Yost index measure scores census tracts based on median household income, median house value, median rent, percent below 150% of poverty line, education, percent working class, and percent unemployed. Census tracts are then categorized into quintiles (1=lowest SES, 5=highest SES).[3,4]

4. Construction of inverse probability of treatment weights (IPTW).

We created IPTWs by fitting a logistic regression model to estimate the probability of each patient living in Philadelphia conditional on their observed covariates (i.e., $\Pr(Z=1|\mathbf{X})$, where Z is a binary variable for patients living in Philadelphia and **X** is a vector of confounders). This is the propensity score. The model was stratified by time so that the distribution of covariates in the control patients resembled that of the Philadelphia patients at any given time. We used the propensity score to calculate average treatment effect among the treated (ATT) weights following the approach outlined by Austin and Stuart.[5] Patients living in Philadelphia received a weight of 1 while patients living in control received a weight equal to $\Pr(Z=1|\mathbf{X})/(1-\Pr(Z=1|\mathbf{X}))$. We recreated weights for each subgroup analysis (i.e., we first restricted the population to the subgroup of interest and then created IPTWs for that specific population).

To explore the possibility of non-linear effects of some predictor variables for our propensity score model, we modeled the 2 continuous variables, age and number of pre-tax visits, using restricted cubic splines, with knots placed at the 10th, 25th, 50th, 75th, and 90th percentiles. We did this in case it led to better model fit for the propensity score models. However, we observed that it led to worse balance of covariates between the Philadelphia and control groups (i.e., the difference in the median age at baseline between Philadelphia and control was greater with the spline model than the simple model). We therefore used the simple model in our analyses.

Supplementary Table 1. Characteristics of the cross-sectional dataset by group and period before and after inverse probability of treatment weighting

	**Unweighted**					**Weighted**				
	Philadelphia		Control		SMD[b]	Philadelphia		Control		SMD[b]
Characteristic[a]	Pre	Post	Pre	Post		Pre	Post	Pre	Post	
Age at BMI measurement (y)	47.0 (34.0, 56.5)	46.4 (33.2, 56.5)	49.7 (37.9, 57.5)	50.1 (37.7, 58.0)	0.148	47.0 (34.0, 56.5)	46.4 (33.2, 56.5)	45.6 (33.4, 55.2)	44.9 (31.7, 55.3)	0.099
Gender										
Male	116,747 (40%)	139,592 (41%)	258,040 (41%)	336,333 (42%)	0.018	116,747 (40%)	139,592 (41%)	116,349 (41%)	140,948 (42%)	0.015
Female	175,338 (60%)	199,142 (59%)	370,710 (59%)	466,399 (58%)		175,338 (60%)	199,142 (59%)	169,592 (59%)	195,153 (58%)	
Race										
White	111,895 (38%)	134,719 (40%)	516,669 (82%)	651,692 (81%)	1.062	111,895 (38%)	134,719 (40%)	115,919 (41%)	137,817 (41%)	0.052
Black	153,560 (53%)	169,896 (50%)	60,336 (10%)	73,525 (9%)		153,560 (53%)	169,896 (50%)	141,775 (50%)	161,593 (48%)	
Asian-American/ Pacific Islander	12,426 (4%)	16,795 (5%)	22,709 (4%)	37,613 (5%)		12,426 (4%)	16,795 (5%)	13,561 (5%)	18,639 (6%)	
Other/multiple	14,204 (5%)	17,324 (5%)	29,036 (5%)	39,902 (5%)		14,204 (5%)	17,324 (5%)	14,687 (5%)	18,051 (5%)	
Hispanic ethnicity	11,544 (4%)	15,070 (4%)	19,478 (3%)	29,680 (4%)	0.041	11,544 (4%)	15,070 (4%)	12,895 (5%)	15,714 (5%)	0.019
Medicaid	63,594 (22%)	80,616 (24%)	24,464 (4%)	34,065 (4%)	0.572	63,594 (22%)	80,616 (24%)	59,337 (21%)	77,783 (23%)	0.020
Yost Quintile										
1	135,592 (46%)	156,189 (46%)	17,407 (3%)	21,822 (3%)	1.784	135,592 (46%)	156,189 (46%)	130,288 (46%)	153,884 (46%)	0.013
2	59,726 (20%)	68,659 (20%)	26,941 (4%)	30,846 (4%)		59,726 (20%)	68,659 (20%)	58,307 (20%)	67,706 (20%)	
3	38,161 (13%)	44,727 (13%)	78,970 (13%)	97.714 (12%)		38,161 (13%)	44,727 (13%)	38,311 (13%)	44,988 (13%)	
4	22,959 (12%)	40,552 (12%)	155,547 (25%)	196,233 (24%)		22,959 (12%)	40,552 (12%)	34,280 (12%)	40,803 (12%)	
5	24,647 (8%)	28,607 (8%)	349,885 (56%)	456,117 (57%)		24,647 (8%)	28,607 (8%)	24,756 (9%)	28,719 (9%)	

[a]Median (interquartile range) or N (%)

[b]The standardized mean difference between Philadelphia and control (i.e., across both pre- and post-tax periods)

Supplementary Table 2. Sensitivity analyses for changes in BMI trends[a] after Philadelphia beverage tax implementation

Analysis	N patients	Control			Philadelphia			Differenced trend change in BMI[c]	P-value[d]
		Baseline BMI[b]	Pre-tax BMI trend	Post-tax BMI trend change	Baseline BMI[b]	Pre-tax BMI trend	Post-tax BMI trend change		
**Panel**									
Aggregate data at patient-month level	175,675	31.6 (31.2, 32.0)	-0.01 (-0.02, 0.00)	0.03 (0.01, 0.04)	30.6 (30.4, 30.8)	0.00 (0.00, 0.01)	0.01 (0.00, 0.01)	-0.02 (-0.04, 0.00)	0.053
Exclude first 6 months post	160,881	31.2 (30.9, 31.6)	0.00 (-0.03, 0.02)	0.04 (0.00, 0.09)	30.4 (30.3, 30.5)	0.02 (0.01, 0.02)	0.02 (0.00, 0.03)	-0.03 (-0.07, 0.02)	0.28
Remove BMI outliers	174,585	30.6 (30.3, 30.9)	-0.01 (-0.04, 0.01)	0.05 (0.01, 0.08)	29.8 (29.7, 29.9)	0.00 (0.00, 0.01)	0.04 (0.02, 0.05)	-0.01 (-0.05, 0.03)	0.61
Include only adults with BMI measures in every year of the study period	36,223	32.0 (31.4, 32.6)	0.04 (0.00, 0.08)	-0.04 (-0.11, 0.02)	31.3 (31.1, 31.4)	0.04 (0.03, 0.05)	-0.03 (-0.05, -0.02)	0.01 (-0.06, 0.08)	0.75
**Cross-sectional sample**									
Aggregate data at patient-month level	587,121	31.0 (30.7, 31.3)	-0.01 (-0.02, 0.00)	0.01 (0.00, 0.03)	30.1 (30.0, 30.2)	0.01 (0.00, 0.01)	-0.01 (-0.01, 0.00)	-0.02 (-0.04, -0.01)	0.0044
Exclude first 6 months	572,143	30.6 (30.4, 30.9)	-0.01 (-0.03, 0.01)	0.03 (-0.01, 0.06)	29.8 (29.7, 29.9)	0.02 (0.01, 0.02)	-0.02 (-0.04, -0.01)	-0.05 (-0.09, -0.01)	0.021
Remove BMI outliers	582,054	30.0 (29.8, 30.2)	-0.01 (-0.03, 0.01)	0.02 (-0.01, 0.05)	29.3 (29.2, 29.3)	0.01 (0.01, 0.02)	-0.01 (-0.02, 0.00)	-0.03 (-0.06, 0.00)	0.047

[a]Models were fit with generalized estimating equations weighted by inverse probability of treatment weights with variables for group (Philadelphia=1, control=0), time (continuous quarter), trend change (i.e., continuous quarter since tax implementation), a group-by-time interaction, a group-by-trend change interaction, and indicator terms for season. We estimated the baseline BMI level and pre-tax trend in BMI in Philadelphia using the same model but reversing the coding of the Philadelphia and control group.

[b]Mean BMI in the first quarter of 2014

[c]Post-tax change in BMI trend in Philadelphia, less that of the control group

[d]P-value for the differenced trend change

Supplementary Table 3. Sensitivity analyses for changes in obesity prevalence trends[a] after Philadelphia beverage tax implementation

Analysis	N patients	Control			Philadelphia				
		Baseline obesity prevalence[b]	Pre-tax trend in obesity prevalence	Post-tax trend change in obesity prevalence	Baseline obesity prevalence[b]	Pre-tax trend in obesity prevalence	Post-tax trend change in obesity prevalence	Differenced trend change in obesity prevalence[c]	P-value[d]
**Panel**									
Aggregate data at patient-month level	175,675	51.1% (48.7, 53.5)	-0.1pp (-0.1, 0.0)	0.2pp (0.1, 0.3)	45.6% (44.6, 46.5)	0.0pp (0.0, 0.0)	0.1pp (0.0, 0.1)	-0.1pp (-0.2, 0.0)	0.035
Exclude first 6 months post	160,881	49.4% (47.5, 51.3)	0.0pp (-0.2, 0.1)	0.3pp (0.1, 0.6)	44.7% (43.9, 45.4)	0.1pp (0.0, 0.1)	0.1pp (0.0, 0.2)	-0.2pp (-0.5, 0.1)	0.15
Remove BMI outliers	174,585	48.3% (46.4, 50.3)	-0.1pp (-0.3, 0.0)	0.4pp (0.2, 0.7)	43.3% (42.5, 44.0)	0.0pp (0.0, 0.1)	0.2pp (0.1, 0.3)	-0.2pp (-0.5, 0.1)	0.15
Include only adults with BMI measures in every year of the study period	36,223	54.1% (51.0, 57.2)	0.1pp (-0.1, 0.4)	-0.1pp (-0.4, 0.3)	49.7% (48.6, 50.8)	0.3pp (0.2, 0.3)	-0.2pp (-0.4, -0.1)	-0.2pp (-0.5, 0.2)	0.43
**Cross-sectional sample**									
Aggregate data at patient-month level	587,121	47.7% (46.2, 49.2)	0.0pp (-0.1, 0.0)	0.1pp (0.0, 0.2)	42.7% (42.0, 43.5)	0.0pp (0.0, 0.1)	0.0pp (-0.1, 0.0)	-0.1pp (-0.2, 0.0)	0.0097
Exclude first 6 months	572,143	45.7% (44.5, 47.0)	0.0pp (-0.2, 0.1)	0.1pp (-0.1, 0.4)	40.9% (40.3, 41.4)	0.1pp (0.0, 0.1)	-0.1pp (-0.2, 0.0)	-0.3pp (-0.5, 0.0)	0.030
Remove BMI outliers	582,054	44.5% (43.2, 45.8)	-0.1pp (-0.2, 0.1)	0.2pp (-0.1, 0.4)	39.8% (39.2, 40.4)	0.1pp (0.0, 0.1)	-0.1pp (-0.2, 0.0)	-0.2pp (-0.5, 0.0)	0.038

[a]Models were fit with generalized estimating equations weighted by inverse probability of treatment weights with variables for group (Philadelphia=1, control=0), time (continuous quarter), trend change (i.e., continuous quarter since tax implementation), a group-by-time interaction, a group-by-trend change interaction, and indicator terms for season. Baseline obesity prevalence is reported as a percent for each location. All trends and trend changes are reported as percentage points (pp). We estimated the baseline obesity prevalence level and pre-tax trend in obesity prevalence in Philadelphia using the same model but reversing the coding of the Philadelphia and control group.

[b]Obesity prevalence in the first quarter of 2014

[c]Post-tax change in obesity prevalence trend in Philadelphia, less that of the control group

[d]P-value for the differenced trend change

Supplementary Table 4. Changes in HbA1c trends[a] after Philadelphia beverage tax implementation

		Control			Philadelphia				
Sample	N patients	Baseline HbA1c[b]	Pre-tax HbA1c trend	Post-tax HbA1c trend change	Baseline HbA1c[b]	Pre-tax HbA1c trend	Post-tax HbA1c trend change	Differenced trend change in HbA1c[c]	P-value[d]
Overall	35,295	5.6 (5.5, 5.6)	0.02 (0.01, 0.02)	0.01 (0.00, 0.02)	5.5 (5.4, 5.5)	0.02 (0.02, 0.03)	0.00 (0.00, 0.00)	-0.01 (-0.02, 0.00)	0.13
Mean pre-tax HbA1c of 5.7-6.4	14,405	5.9 (5.8, 5.9)	0.01 (0.00, 0.02)	0.01 (0.00, 0.03)	5.8 (5.8, 5.8)	0.02 (0.01, 0.02)	0.01 (0.01, 0.02)	0.00 (-0.02, 0.01)	0.79

[a]Models were fit with generalized estimating equations weighted by inverse probability of treatment weights with variables for group (Philadelphia=1, control=0), time (continuous quarter), trend change (i.e., continuous quarter since tax implementation), a group-by-time interaction, a group-by-trend change interaction, and indicator terms for season. We estimated the baseline HbA1c level and pre-tax trend in HbA1c in Philadelphia using the same model but reversing the coding of the Philadelphia and control group.

[b]Mean HbA1c in the first quarter of 2014

[c]Post-tax change in HbA1c trend in Philadelphia, less that of the control group

[d]P-value for the differenced trend change

Supplementary Figure 1. Directed acyclic graph for the association between the Philadelphia beverage tax and change in adult BMI

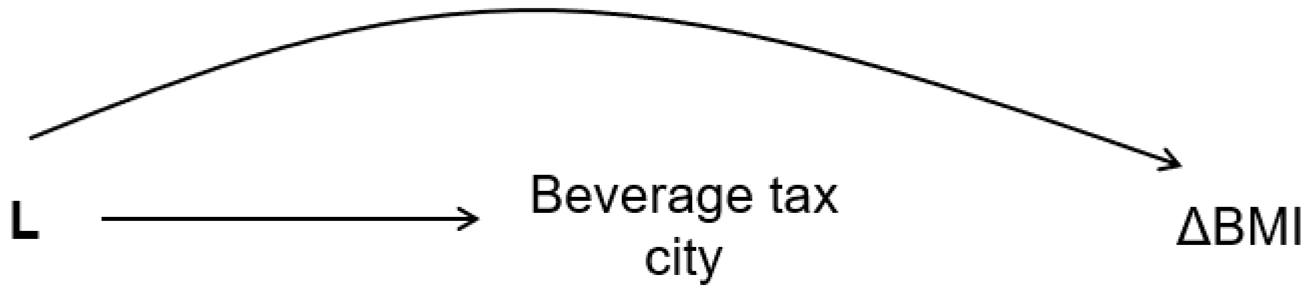

This DAG illustrates the need to adjust for factors (**L**) that are associated with living in Philadelphia (the beverage tax city) and with change in BMI, which could confound associations. **L** includes age, gender, race, Hispanic ethnicity, Medicaid status, Yost score, and healthcare utilization, all of which may be associated with city and with pre-post changes in BMI.

Supplementary Figure 2. Differences in the distribution of BMI from pre- to post-tax for Philadelphia and control patients (weighted by IPTWs)

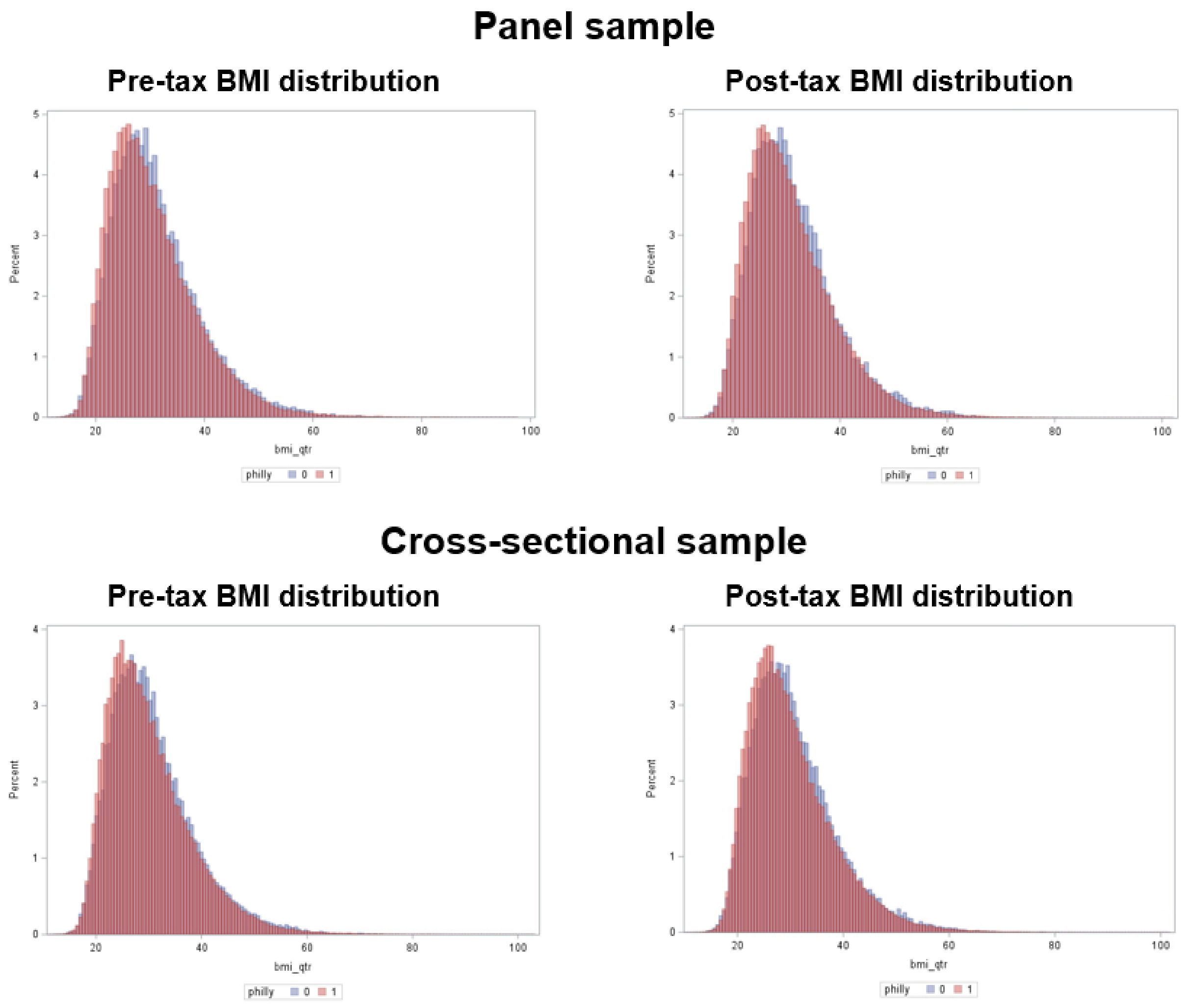

REFERENCES

1. Daymont C, Ross ME, Russell Localio A, Fiks AG, Wasserman RC, Grundmeier RW. Automated identification of implausible values in growth data from pediatric electronic health records. *J Am Med Inform Assoc JAMIA*. 2017;24(6):1080-1087. doi:10.1093/jamia/ocx037

2. Lin PID, Rifas-Shiman SL, Aris IM, et al. Cleaning of anthropometric data from PCORnet electronic health records using automated algorithms. *JAMIA Open*. 2022;5(4):ooac089. doi:10.1093/jamiaopen/ooac089

3. Yost K, Perkins C, Cohen R, Morris C, Wright W. Socioeconomic status and breast cancer incidence in California for different race/ethnic groups. *Cancer Causes Control CCC*. 2001;12(8):703-711. doi:10.1023/a:1011240019516

4. Yu M, Tatalovich Z, Gibson JT, Cronin KA. Using a composite index of socioeconomic status to investigate health disparities while protecting the confidentiality of cancer registry data. *Cancer Causes Control CCC*. 2014;25(1):81-92. doi:10.1007/s10552-013-0310-1

5. Austin PC, Stuart EA. Moving towards best practice when using inverse probability of treatment weighting (IPTW) using the propensity score to estimate causal treatment effects in observational studies. *Stat Med*. 2015;34(28):3661-3679. doi:10.1002/sim.6607
